# Regulation of cardiac fibroblast-mediated maladaptive ventricular remodeling by β-arrestins

**DOI:** 10.1371/journal.pone.0219011

**Published:** 2019-07-03

**Authors:** Jennifer L. Philip, Xianyao Xu, Mei Han, Shahab A. Akhter, Md Abdur Razzaque

**Affiliations:** 1 Department of Surgery, Division of Cardiothoracic Surgery, University of Wisconsin School of Medicine and Public Health, Madison, Wisconsin, United States of America; 2 Department of Surgery, Section of Cardiac and Thoracic Surgery, The University of Chicago Medical Center, Chicago, Illinois, United States of America; 3 Department of Cardiovascular Sciences, East Carolina Heart Institute at East Carolina University, Greenville, North Carolina, United States of America; 4 Department of Surgery and Cardiovascular Center of Excellence, Louisiana State University, New Orleans, Louisiana, United States of America; University of Cincinnati College of Medicine, UNITED STATES

## Abstract

Cardiac fibroblasts (CF) play a critical role in post-infarction remodeling which can ultimately lead to pathological fibrosis and heart failure. Recent evidence demonstrates that remote (non-infarct) territory fibrosis is a major mechanism for ventricular dysfunction and arrhythmogenesis. β-arrestins are important signaling molecules involved in β-adrenergic receptor (β-AR) desensitization and can also mediate signaling in a G protein independent fashion. Recent work has provided evidence that β-arrestin signaling in the heart may be beneficial, however, these studies have primarily focused on cardiac myocytes and their role in adult CF biology has not been well studied. In this study, we show that β-arrestins can regulate CF biology and contribute to pathological fibrosis. Adult male rats underwent LAD ligation to induce infarction and were studied by echocardiography. There was a significant decline in LV function at 2–12 weeks post-MI with increased infarct and remote territory fibrosis by histology consistent with maladaptive remodeling. Collagen synthesis was upregulated 2.9-fold in CF isolated at 8 and 12 weeks post-MI and β-arrestin expression was significantly increased. β-adrenergic signaling was uncoupled in the post-MI CF and β-agonist-mediated inhibition of collagen synthesis was lost. Knockdown of β-arrestin1 or 2 in the post-MI CF inhibited transformation to myofibroblasts as well as basal and TGF-β-stimulated collagen synthesis. These data suggest that β-arrestins can regulate CF biology and that targeted inhibition of these signaling molecules may represent a novel approach to prevent post-infarction pathological fibrosis and the transition to HF.

## Introduction

Heart failure (HF) is a frequent complication of myocardial infarction (MI) that is associated with adverse ventricular remodeling [1]. As a result of improved survival post-MI, more people are at risk of developing HF. Within 6 years of the clinical event of an acute MI, 22% of male and 46% of female patients develop HF [2]. Well-healed infarcts contain large amounts of extracellular matrix (ECM) proteins which can occupy up to 80% of the infarct area [3]. Importantly, collagen deposition also occurs in the non-infarcted remote myocardium, predominantly in the interstitium, where it contributes to ventricular stiffness and dysfunction [4].

Cardiac fibroblasts (CF) make up 60–70% of the total cell number of the heart and play a critical role in regulating normal myocardial function as well as in the adverse remodeling that can occur post-MI and the transition to HF. CF are responsible for ECM deposition and create the scaffold for cardiac myocytes [5]. Cardiac fibrosis is characterized by overproduction of ECM proteins, predominantly collagen types I and III, in the interstitial and perivascular space [6]. Excessive collagen deposition leads to myocardial stiffening, impaired cardiac relaxation and filling (diastolic dysfunction), and overload of the heart. This increased fibrosis is associated with the transformation of quiescent fibroblasts, responsible for basal ECM homeostasis, to activated myofibroblasts, which are characterized by increased expression of α-smooth muscle actin (α-SMA), collagen I, III, and IV, and fibronectin [7]. Novel mechanisms of regulation of CF to myofibroblast transformation are just beginning to be understood. No targeted therapies currently exist to prevent myofibroblast formation and excessive collagen deposition in the non-infarcted remote myocardium which is a major etiology for the development of HF.

Recent studies have demonstrated that increased intracellular cAMP in CF can prevent transformation to myofibroblasts *in vitro* [8, 9]. Cyclic AMP production in CF is stimulated by signaling through β_2_-adrenergic receptors (β_2_-ARs) which couple to adenylyl cyclase. The role of β-arrestin signaling in CF is just beginning to be understood. β-arrestins are ubiquitous G-protein-coupled receptor adaptor proteins that negatively regulate cardiac β-AR function via receptor desensitization and downregulation. In addition, they mediate G protein-independent signaling which may be beneficial in the heart. Recent data from our lab demonstrated increased β-arrestin expression in CF isolated from failing human left ventricles [10]. Arrestin signaling was shown to be important in regulating fibroblast transformation and collagen synthesis via β-AR/cAMP and TGF-β/smad-dependent pathways.

In this study, we investigate the role of β-arrestins in regulating CF biology in fibroblasts isolated from adult rat left ventricles following myocardial infarction with a focus on therapeutic inhibition of β-arrestin signaling.

## Materials and methods

All cell culture reagents were purchased from Invitrogen Technologies (Eugene, OR) except fetal bovine serum (FBS), obtained from Atlanta Biologicals (Lawrenceville, GA). Unless stated otherwise, all additional chemicals were obtained from Sigma-Aldrich (St. Louis, MO). All antibodies were obtained from Santa Cruz Biotechnology (Santa Cruz, CA) except α-SMA and vimentin antibodies were obtained from Sigma-Aldrich (St. Louis, MO), Collagen types I&III and β-arrestin1&2 from Abcam (Cambridge, MA). anti-β-arrestin1 (Abcam ab32099,1:1000), anti-Collagen I (Abcam ab34710, 1:1000), α-Tubulin (Santa Cruz sc-32293, 1:1000) and GAPDH (Santa Cruz sc-25778, 1:1000) were used for immunoblotting. anti-β-arrestin1 (Abcam ab32099,1:200), anti-β-arrestin2 (Abcam ab31294, 1:200), anti-Collagen I (Abcam ab34710, 1:500), anti-Collagen III (Abcam ab6310, 1:200), anti-fibronectin (Santa Cruz sc-9068, 1:500), and vimentin (sc-5565, 1:400) antibodies were used for immunostaining.

### Animal procedures and rat myocardial infarction model

Experiments involving animals were approved by the Institutional Animal Care and Use Committee of the University of Chicago and University of Wisconsin, and in accordance with the National Institutes of Health Guidelines for the care and use of laboratory animals. Myocardial infraction (MI) was induced as described previously using a left anterior descending coronary artery ligation that produces a highly reproducible infarction [11, 12]. Briefly, anesthetized Sprague-Dawley adult male rats were intubated and mechanically ventilated. Anesthesia was maintained using a 2% isoflurane (v/v) oxygen mixture. Rats were placed in the left lateral decubitus position and the chest cavity was opened through a left thoracotomy at the third intercostal space to achieve visualization of the heart. The pericardium was opened. MI was induced through permanent ligation of the LAD coronary artery with a 8–0 silk suture proximal to its bifurcation from the main stem. LV blanching indicated successful occlusion of the LAD. The incision was subsequently closed with 5–0 silk suture, and the rats were allowed to recover. Animals were sacrificed 2, 4, 8, or 12 weeks post-MI surgery (n = 10 for 2 and 4 week groups, n = 20 for 8 and 12 week groups). Control rats did not undergo surgery (n = 14).

### Echocardiography

Echocardiography was performed at 2, 4, 8, and 12 weeks post-MI to assess global cardiac function as previously described [13]. VisualSONICS Vevo 2100 (Visual Sonics Inc., Toronto, Ontario, Canada) imaging system with a 250 scanhead in anesthetized animals (2% isoflurane, v/v). The internal diameter of the LV was measured in the short-axis view from M-mode recordings in end diastole and end systole. Vevo 2100 Imaging System analysis software was used to calculate ejection fraction (EF) and fractional shortening (FS) using the formulas as previously described [14].

### Histology

The left ventricles (LV) of control and post-MI rats were fixed in 4% paraformaldehyde, embedded with paraffin, and cut cross-sectionally into 5-μm-thick sections along the center of the fibrotic scar. Masson trichrome and picrosirius red staining was used to evaluate cardiac fibrosis and collagen deposition. Sections were imaged at 20x magnification by bright-field microscopy (CRi Panoramic scan whole slide scanner using Zeiss AxioCam MRm, Germany). The quantity of cardiac fibrosis was assessed by calculating percent fibrotic area by modification of the previously described method of Pan et al [15]. Percent fibrotic area was determined as the ratio of collagen surface area stained blue by trichrome or red by picrosirius red staining to the total myocardial surface area. Five areas were selected at random from the remote (non-infarct) territories of each heart and values were averaged for each rat heart over all five sections. Infarct size was assessed by examining images obtained at low magnification and calculated as the ratio of scar area to total LV cross sectional area. All quantitative evaluations were carried out by NIH ImageJ software.

### Isolation and culture of adult rat cardiac fibroblasts

Cardiac fibroblasts were isolated from the LV of either control or post-MI adult Sprague-Dawley rats using previously described methods [16–19]. Briefly, rat left ventricles were minced and digested with 100 U/mL collagenase and 0.6 mg/mL trypsin, isolated fibroblasts were plated on a non-coated dish in Dulbecco’s modified Eagle’s medium containing 10% fetal bovine serum and 100 U/mL penicillin, 100 μg/mL streptomycin, and 0.25 μg/mL amphotericin B (Antibiotic-Antimycotic, Life Technologies, Carlsbad, CA). To prevent spontaneous differentiation, all studies were carried out in low serum (2.5% FBS) medium using early passage cells (≤3) plated at a density of ~200 cells per mm^2^. Post-MI CF were used within two weeks of culturing to ensure preservation of the phenotype.

### Protein immunoblotting

Cells were lysed in buffer containing 25 mM HEPES, 1 mM EDTA, 125 mM NaCl, .5 mM NaF, .25% Nonidet P-40, 5% glycerol (pH 6.8), 10 μg/ml leupeptin, 20 μg/ml aprotinin and 1mM phenyl-methylsulfonyl fluoride. Equal amounts of protein for each sample were separated by SDS-PAGE, transferred onto a nitrocellulose membrane, and immunoblotted. Bands were visualized with ECL Western blotting substrate (Thermo Scientific, Rockford, IL). Band intensity was quantitated using NIH ImageJ software. GAPDH or α-tubulin was used as a loading control.

### siRNA transfection of cardiac fibroblasts

Target-specific siRNA duplexes were designed using the sequence from the open reading frame of rat β-arrestins to knockdown mRNA and protein expression of β-arrestins. β-arrestin1 (sc-29741, 5′-AAAGCCUUCUGCGCGGAGAAU-3′) and β-arrestin2 (sc-29208, 5′-AAGGACCGCAAAGUGUUUGUG-3′) siRNA were obtained from Santa Cruz Biotechnology. Scrambled oligo-ribonucleotide complex was also obtained (sc-37007), which was not homologous to any mammalian genes and utilized as control. Cells were transfected with Lipofectamine 2000 (Invitrogen; Carlsbad, CA), according to manufacturers’instructions. Silencing was confirmed by immunofluorescence. Only experiments with verified silencing were used.

### Intracellular cAMP quantitation

CF at 80% confluence cultured on 12-well plates were equilibrated for 12 h in low-serum (2.5% FBS) DMEM and assayed for intracellular cAMP accumulation by a 15-min incubation with 0.2 mM isobutylmethylxanthine, a cyclic nucleotide phosphodiesterase inhibitor, followed by addition of ISO (10μM) for an additional 15 min. Reactions were terminated by aspiration of culture medium and addition of 150μl of 0.1 M HCl to each well. HCl extracts were assayed for cAMP content by direct ELISA kit (Assay Designs, Ann Arbor, MI).

### Drug treatment protocol

CF were grown to desired confluence in supplemented DMEM and treated with either transforming growth factor-β (TGF-β) to reach a final concentration of 10ng/mL, β-agonist Isoproterenol (ISO) to reach a final concentration of 10μM, or no drug in DMEM with 2.5% FBS. Cells were collected following 24 hours or 72 hours.

### Collagen synthesis by [^3^H]proline incorporation

[^3^H]proline incorporation was measured according to the method of Swaney et al [9]. Cells were grown to 80% confluence on 12-well plates, serum starved for 12hrs, and incubated with [^3^H]proline (1 μCi/well, Perkin Elmer Life Sciences, Shelton, CT, USA) for 48 hours in either 2.5% FBS with 10ng/mL TGF-β, 10μM ISO, or no drug. Cellular protein was precipitated overnight with 20% trichloroacetic acid (TCA) and washed 3 times with 1ml of 5% TCA plus 0.01% proline, then dissolved in 0.2 M NaOH. The activity of [^3^H]proline was determined by liquid scintillation counting.

### Immunostaining and confocal microscopy

CF cells were grown to 60% confluence on 12 mm coverslips, washed with PBS, and fixed with 3.7% formaldehyde for 15 min. Cells were washed and then blocked for 2 hours in PBS with 10% FBS and 0.3% Triton X-100. After several washes with PBS + 0.3% Triton X-100, cells were treated using the following primary antibodies (and dilutions) overnight at 4°C: α-SMA, Vimentin, Collagen I, Collagen III, β-arrestin1&2 and Fibronectin. This was followed by treatment with secondary antibody for 2 hours (All secondary antibodies were purchased from Invitrogen Molecular Probes, Eugene, OR, USA). After three washes with PBS +0.3% Triton X-100, cells were mounted in Fluoroshield with DAPI histology mounting medium from Sigma-Aldrich (St. Louis, MO, USA). Cells were mounted in Fluoroshield with DAPI mounting medium (Sigma-Aldrich) and visualized using an Olympus DSU Spinning Disk Confocal microscope.

### Statistical analysis

All data are expressed as mean ± SEM. cAMP quantitation was done using GraphPad Prism 5 (GraphPad Software, San Diego, CA, USA). Student’s t-test, one-way or two-way ANOVA followed by Tukey’s HSD post hoc test were used. Values of p<0.05 were considered significant.

## Results

### Cardiac dysfunction and remote territory fibrosis develop early post-myocardial infarction

**Fig 1:**
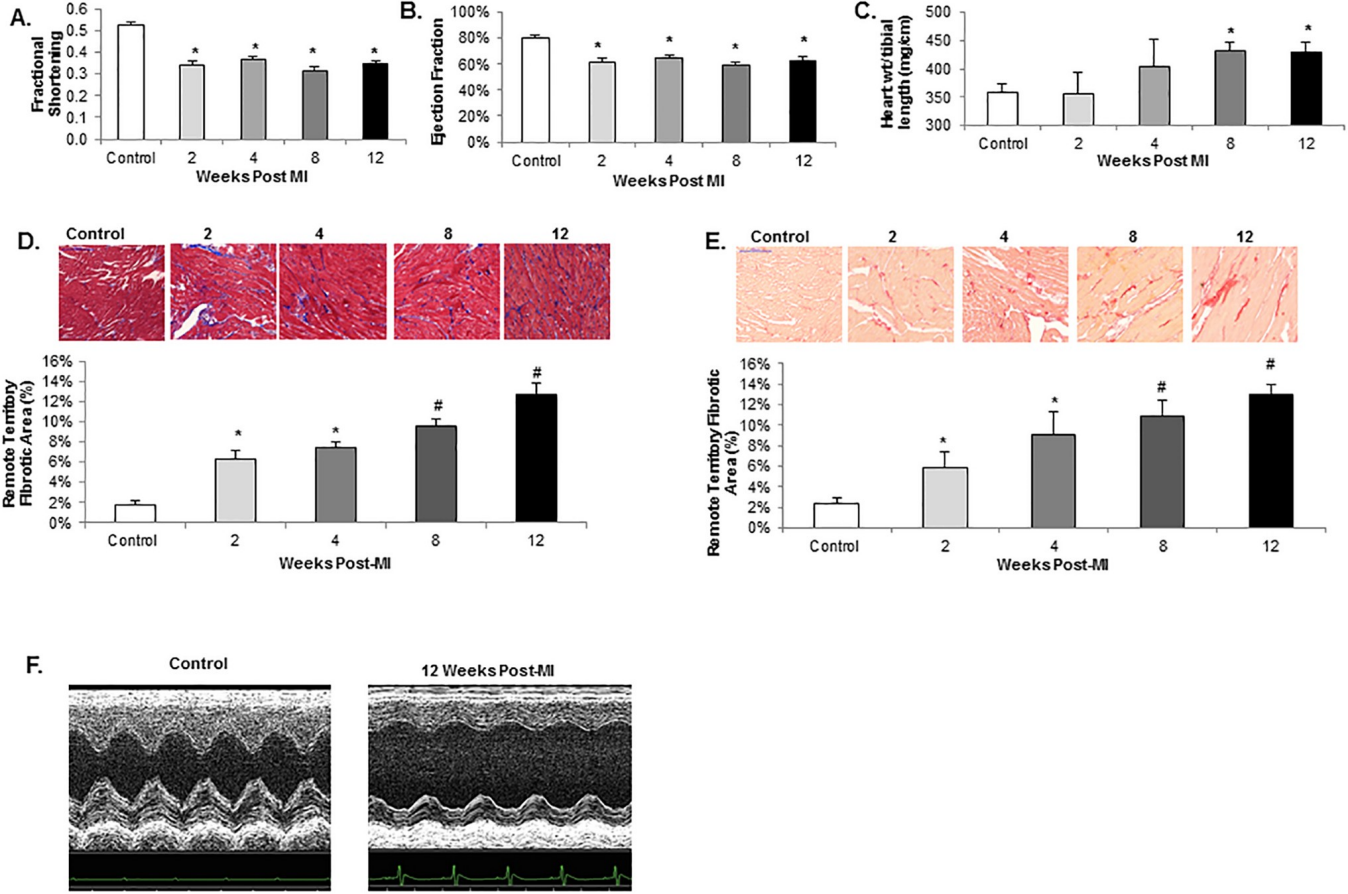
Development of post-infarction ventricular dysfunction and myocardial fibrosis. **A.** Fractional shortening 2–12 weeks post-myocardial infarction (MI) vs. control showing early development of cardiac dysfunction that is sustained through 12 weeks. *p<0.0001 vs. control; n = 4–6 in each group. **B.** Ejection Fraction 2–12 weeks post-MI vs. control. *p<0.001 vs. control; n = 4–6 in each group. **C.** Heart weight to tibial length ratio post-MI vs. control showing development of significant cardiac hypertrophy by 8 weeks. *p<0.025 vs. control; n = 3–6 in each group. **D.** Representative photomicrographs of Masson trichrome stained remote (non-infarct) territories of left ventricles (20x) *(above)* 2–12 weeks post-MI vs. control. Quantitation of percent fibrotic area (purple/blue staining area) in remote territories of left ventricle shown below. *p<0.03 vs. control, #p<0.02 vs. control and vs. 2 and 4 weeks post-MI; n = 3–8 in each group. **E.** Representative photomicrographs of picrosirius red stained remote (non-infarct) territories of left ventricles (20x) *(above)* 2–12 weeks post-MI vs. control. Quantitation of percent fibrotic area (red staining area) in remote territories of left ventricle shown below. *p<0.04 vs. control, ^#^p<0.01 vs. control and vs. 2 weeks post-MI; n = 3–8 in each group. **F.** M-mode echocardiography of control and 12 weeks post-MI rats.

Cardiac function measured by echocardiography was significantly reduced at 2 weeks post-myocardial infarction (MI) as evidenced by a 35% decrease in fractional shortening (Fig 1A) and 23% decrease in ejection fraction (Fig 1B). This depressed cardiac function persisted through 12 weeks post-MI (Fig 1A and 1B). There was development of significant cardiac hypertrophy by 8 and 12 weeks post-MI with a 20% increase in the ratio of heart weight to tibial length (Fig 1C). Cardiac fibrosis was determined by histology using both Masson’s trichrome and picrosirius red staining. There was significantly increased fibrosis in the remote, non-infarct territory, as early as two weeks post-MI as demonstrated by a nearly 3-fold increase in fibrotic area by picrosirius red staining and a 3.3-fold increase in fibrotic area by trichrome staining compared to control (Fig 1D and 1E). Remote territory fibrosis increased to 12.6% at 12 weeks post-MI compared to 1.8% in controls as measured by trichrome staining (Fig 1D). Picrosirius red staining showed similar progressive increases in remote territory fibrosis from 2 through 12 weeks post-MI (Fig 1E). M-mode echocardiography of control and 12 weeks post-MI are shown (Fig 1F).

### Cardiac fibroblasts undergo transformation early after myocardial infarction and have increased expression of β-arrestin1

**Fig 2:**
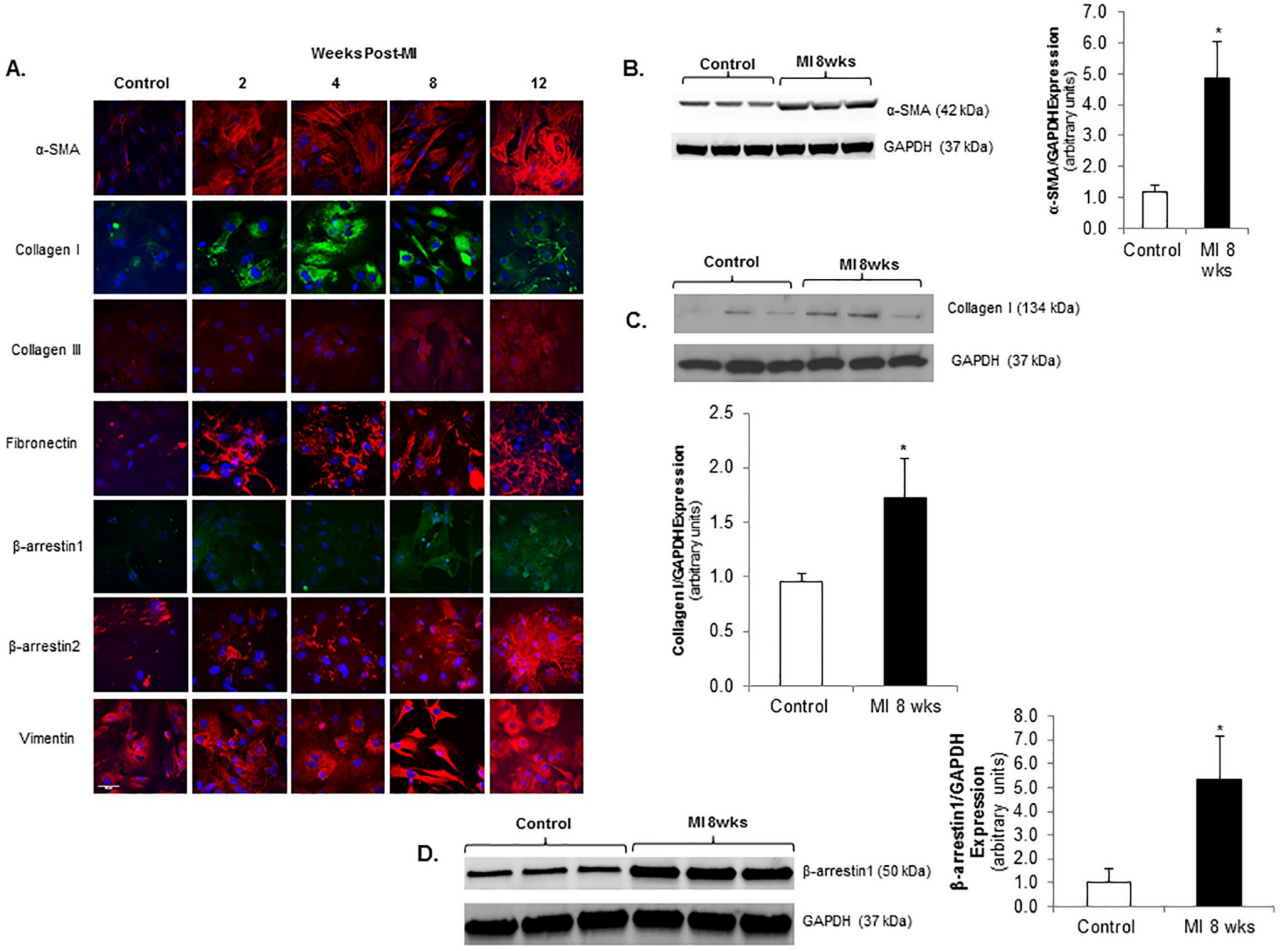
Cardiac fibroblasts undergo early transformation following myocardial infarction with upregulation of β-arrestin 1. **A.** Confocal images of α-SMA, collagen III, fibronectin, β-arrestin 1, β-arrestin 2 and vimentin stained *red* with Alexa Fluor 594 and collagen I stained green with FITC are shown in cardiac fibroblasts isolated from rat LV 2–12 weeks post-MI vs. normal control CF. Nuclei stained *blue* with DAPI. **B.** Representative immunoblots showing increased α-SMA expression in CF isolated 8 weeks post-MI vs. control. This membrane was stripped and re-probed with GAPDH. GAPDH used as loading control. Densitometric analysis showing increased CF α-SMA expression post-MI. *p<0.025 vs. control; n = 4 in each group. **C.** Representative immunoblots showing increased collagen I expression in CF isolated 8 weeks post-MI vs. control. Membrane was cut based on the molecular weight and separately probed with Collagen I and GAPDH. GAPDH used as loading control. Densitometric analysis showing increased CF collagen I expression post-MI. *p<0.025 vs. control; n = 4 in each group. **D.** Representative immunoblots (*upper panel*) showing increased β-arrestin 1 expression in CF isolated 8 weeks post-MI vs. control. This membrane was stripped and re-probed with GAPDH. GAPDH used as loading control. Densitometric analysis (*lower panel*) showing increased CF β-arrestin1 expression post-MI. *p<0.035 vs. control; n = 4 in each group.

Left ventricular fibroblasts were isolated 2, 4, 8, and 12 weeks post-MI. Alpha-smooth muscle actin (α-SMA), a marker of myofibroblast differentiation and CF activation, is markedly elevated at 2 weeks post-MI and remains elevated at 12 weeks (Fig 2A). Collagen I, collagen III, fibronectin and β-arrestin1 and 2 expression are all upregulated as early as 2 weeks post-MI compared to normal control rat CF (Fig 2A). Immunoblotting further showed a highly significant 5-fold increase in α-SMA (p<0.03) and a 2-fold increase in collagen I expression (p<0.03) post-MI, consistent with the pro-fibrotic CF phenotype (Fig 2B and 2C). We measured β-arrestin1 expression specifically in isolated post-MI CF. These CF demonstrated significant upregulation of β-arrestin1 expression compared to normal controls as determined by immunoblotting (5-fold increase, p<0.04, Fig 2D). These data demonstrate upregulation of β-arrestin expression specifically in post-MI CF which have an activated, myofibroblast phenotype known to promote adverse remodeling.

### Myocardial β-arrestin1 expression is upregulated at 8 weeks post-infarction

**Fig 3:**
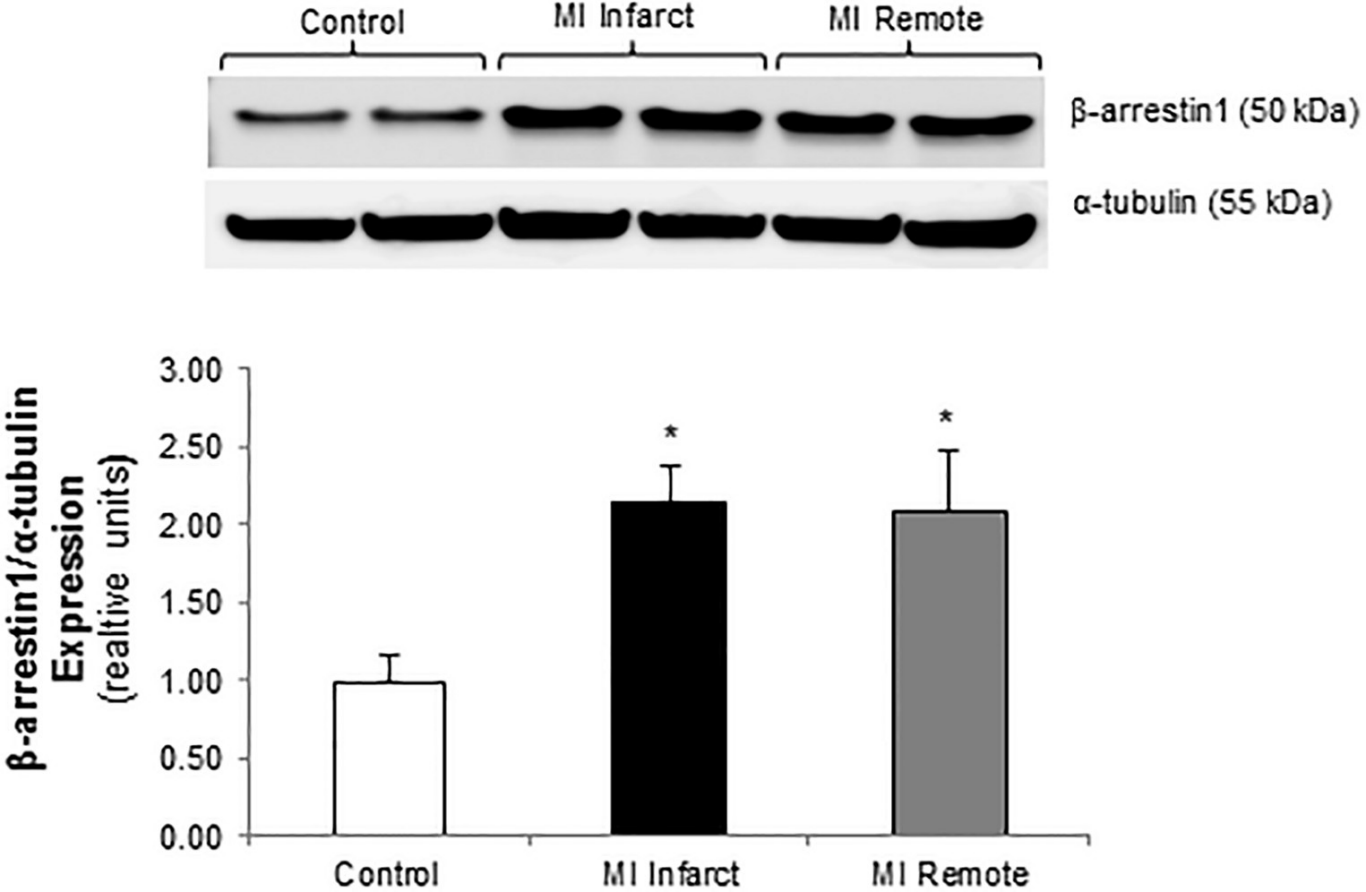
β-arrestin 1 expression is upregulated at 8 weeks post-myocardial infarction. Representative immunoblots (*upper panel*) showing β-arrestin1 expression in the infarct and remote territories of rat left ventricles (LV) 8 weeks post-MI vs. control LV. This membrane was stripped and re-probed with α-tubulin. α-tubulin used as loading control. Densitometric analysis shown below. *p<0.03 vs. control; n = 4 in each group.

**Fig 4:**
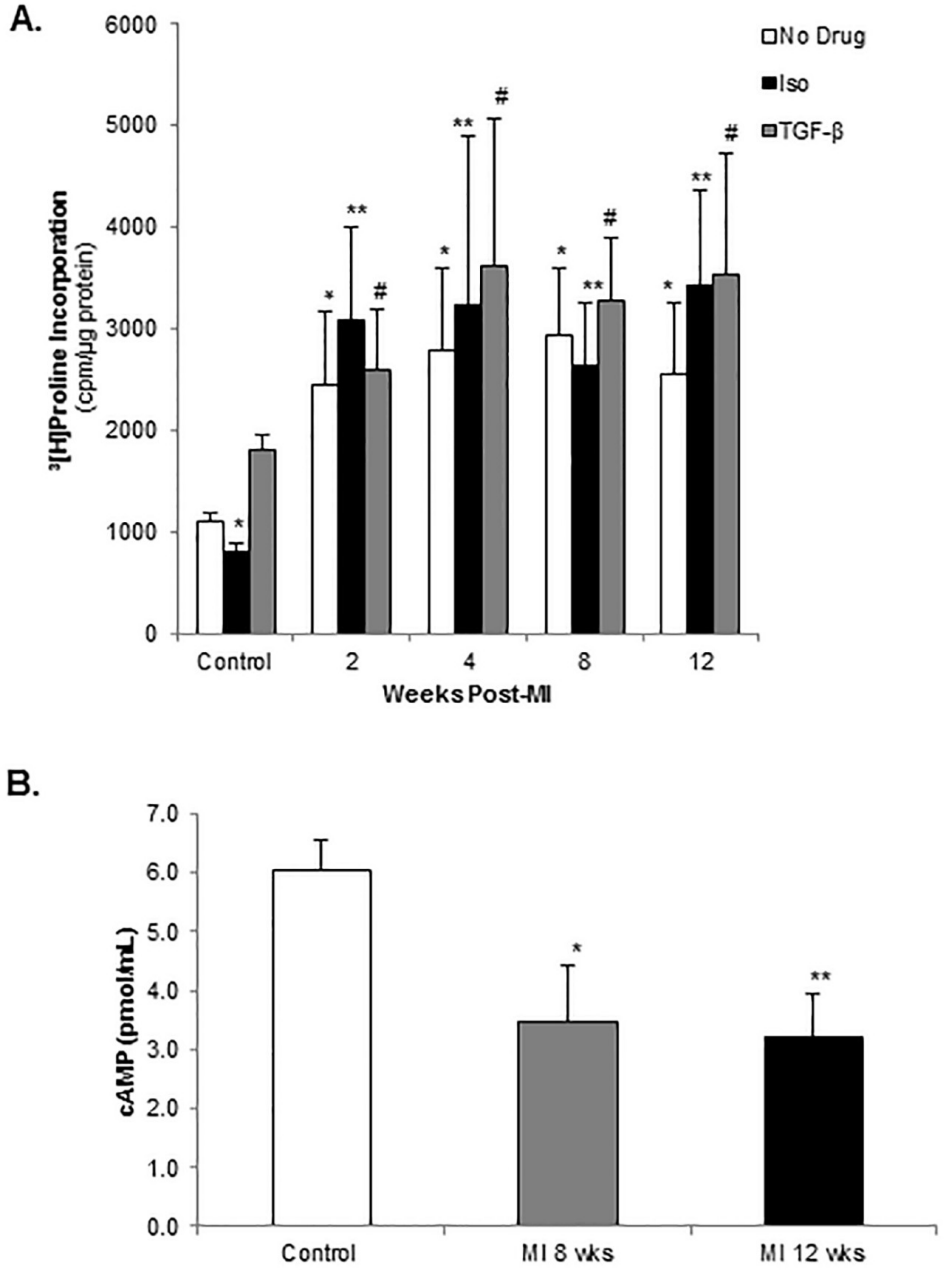
Collagen synthesis and post-MI uncoupling of cardiac fibroblast β-adrenergic signaling. **A.** Collagen synthesis in control and post-MI CF under basal conditions and following TGF-β or β-agonist (Isoproterenol) stimulation. *p<0.02 vs. control + No Drug, **p<0.005 vs. control + Iso, #p<0.01 vs. control + TGF-β; n = 3–9 in each group. **B.** Intracellular cAMP levels in CF isolated 8 and 12 weeks post-MI vs. control. *p = 0.05 vs. control, **p<0.02 vs. control; n = 3 in each group.

We examined β-arrestin1 expression in 8-week post-MI rat LV tissue and there was a 2-fold increase in both the infarct and remote territories compared to control (Fig 3). To study the potential impact of upregulation of β-arrestin1 on isolated CF biology, collagen synthesis was measured by [^3^H]proline incorporation. Normal control CF demonstrated a low level of basal collagen synthesis and a robust increase in response to TGF-β stimulation (Fig 4A). Control CF showed a 40% decrease in collagen synthesis in response to β-agonist stimulation with isoproterenol (Iso) (Fig 4A). This effect was lost in the post-MI CF at 2 weeks and persisted through 12 weeks post-MI. At 8 and 12 weeks post-MI, isolated CF demonstrated a significant decrease in basal intracellular cAMP levels compared to normal control CF (Fig 4B).

### Role of β-arrestin signaling in cardiac fibroblast transformation and ECM synthesis

**Fig 5:**
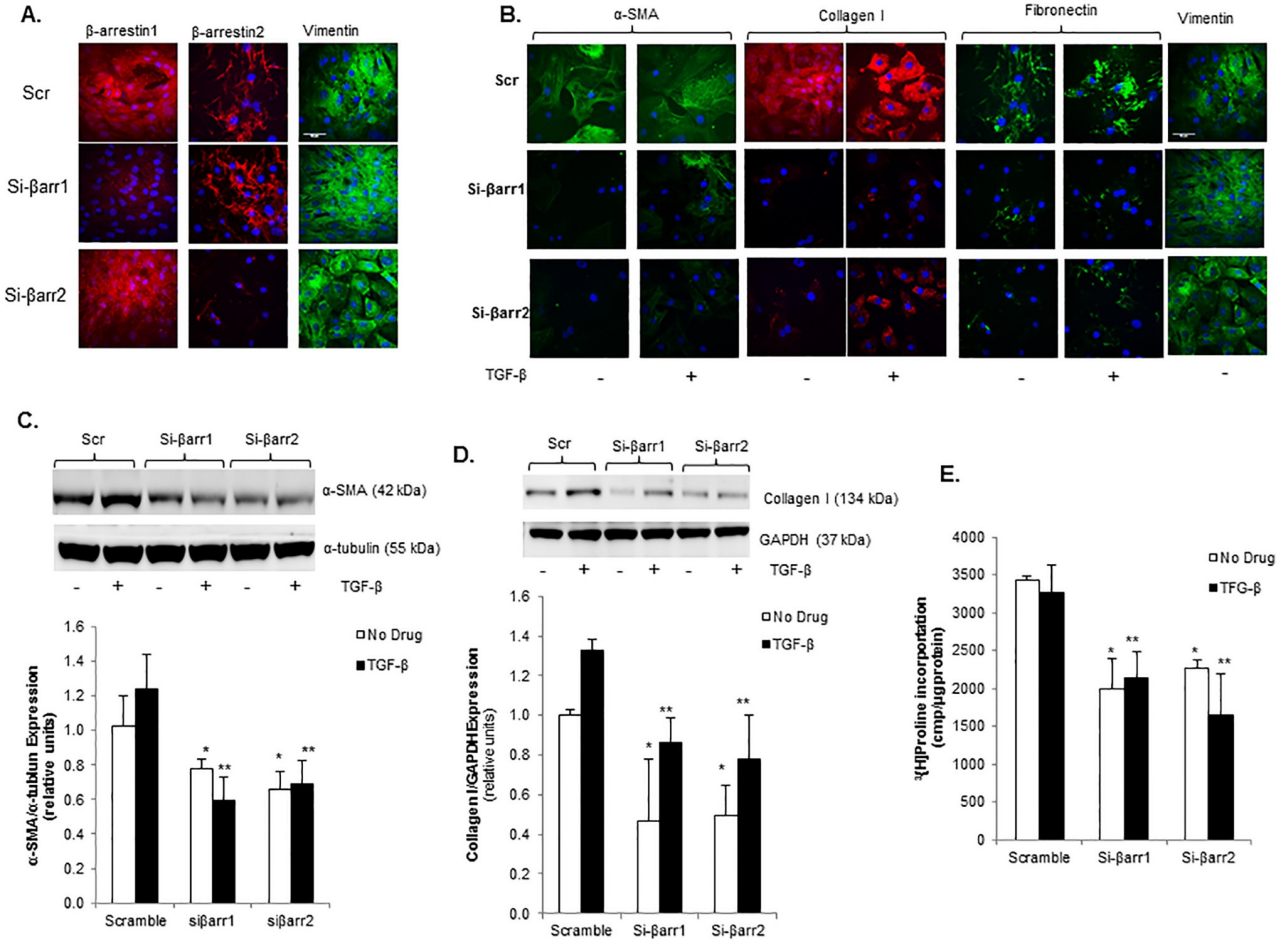
β-arrestin knockdown inhibits myofibroblast transformation and collagen synthesis in cardiac fibroblasts isolated post-myocardial infarction. **A.** Confocal images showing β-arrestin1 and 2 stained red with Alexa Fluor 594 dye and vimentin stained green with FITC in rat cardiac fibroblasts isolated 8 weeks post-infarction treated with siRNA for β-arrestin1 (si-βarr1), β-arrestin2 (si-βarr2), or scrambled control (Scr) demonstrating successful knockdown of β-arrestins. Nuclei stained blue with DAPI. **B.** Confocal images showing Collagen I stained red with Alexa Fluor 594 dye and α-SMA, Fibronectin, and Vimentin stained green with FITC in 8 week post-MI CF following siRNA-mediated knockdown of β-arrestin1 and 2 treated with TGF-β vs. no drug. Nuclei stained blue with DAPI. **C.** Representative immunoblot (upper panel) showing decreased basal and TGF-β-stimulated α-SMA expression in 8 week post-MI CF following Si-βarr1 and Si-βarr2 treatment vs. scramble control (Scr). This membrane was stripped and re-probed for α-tubulin. Densitometric analysis shown below. *p<0.025 vs. Scr No Drug, **p<0.01 vs. Scr TGF-β; n = 4–5 in each group. **D.** Representative immunoblot (upper panel) showing decreased basal and TGF-β-stimulated Collagen I expression in 8 week post-MI CF following Si-βarr1 and Si-βarr2 treatment vs. scramble control (Scr). Membrane was cut based on the molecular weight and separately probed with Collagen I and GAPDH. Densitometric analysis shown below. *p<0.04 vs. Scr No Drug, **p<0.005 vs. Scr No Drug; n = 4–5 in each group. E. Collagen synthesis in 8 week post-MI CF following siRNA knockdown of β-arrestin1 and 2 under basal conditions and TGF-β stimulation. *p<0.02 vs. Scr No Drug, **p<0.02 Scr TGF-β; n = 3 in each group.

To more specifically investigate the role of β-arrestins in post-MI CF transformation and collagen synthesis, β-arrestin1 or 2 expression was inhibited using an siRNA approach in 8 week post-MI isolated CF. Fig 5A demonstrates successful knockdown of β-arrestin1 or 2 (si-βarr1 and si-βarr2, respectively) compared to scrambled siRNA control (Scr). Knockdown of β-arrestin1 resulted in a significant decrease in both basal and TGF-β stimulated α-SMA expression as demonstrated by immunofluorescence and immunoblotting (Fig 5B and 5C). Consistent with lower α-SMA expression and decreased myofibroblast transformation, knockdown of either β-arrestin1 or 2 resulted in significantly lower basal and TGF-β stimulated collagen expression, as measured by immunofluorescence (Fig 5B), immunoblotting (Fig 5D), and collagen synthesis as measured by [^3^H]Proline incorporation (Fig 5E). Knockdown of β-arrestins also had similar effects on fibronectin expression, a marker of ECM production and fibrosis (Fig 5B). These data demonstrate that inhibition of either β-arrestin1 or 2 can reverse the pro-fibrotic phenotype that is characteristic of CF in the post-MI setting and appears to be independent of β-AR/cAMP signaling.

## Discussion

A primary finding of this study is that expression of β-arrestins is upregulated early following myocardial infarction in both the infarct and remote (non-infarct) territories of the left ventricle. This is temporally associated with CF transformation to activated myofibroblasts and increased collagen synthesis and deposition. Specific inhibition of β-arrestin1 or β-arrestin2 expression using an siRNA approach led to inhibition or reversal of the myofibroblast phenotype and decreased collagen and fibronectin expression and collagen synthesis. These effects occurred under basal conditions where the post-MI CF had a very robust elevation in collagen expression compared to non-infarct control CF. In addition, knockdown of β-arrestins also inhibited TGF-β-stimulated α-SMA and collagen I expression and collagen synthesis. This is consistent with our previous work in isolated adult human CF showing that this is mediated through inhibition of MAPK/Smad signaling [10].

Recent work has shown that cAMP can have an antifibrotic action in CF through multiple intracellular signaling pathways. One is the inhibition of Smad-mediated transcription via competition between cAMP response element binding protein (CREB) and Smad for key transcriptional co-activators [9]. Another is cAMP-mediated inhibition of non-Smad signaling pathways activated by TGF-β, including ERK1/2 and JNK MAP kinases. These and other antifibrotic actions of cAMP appear to occur through activation of both PKA and exchange protein activated by cAMP-1 (Epac-1) [20].

Based on recent work from our group and others, another important mechanism may be inhibition of β-arrestin signaling. We have shown that β-arrestin overexpression in normal adult human cardiac fibroblasts leads to increased basal collagen synthesis that is mediated through ERK and Smad signaling [11]. This was abolished by treatment with an ERK inhibitor. Knockdown of β-arrestins in these failing CF, where β-arrestin expression is increased more than 2-fold, using a siRNA approach decreased α-SMA expression and inhibited collagen and fibronectin synthesis. In the current study, β-arrestin expression is upregulated early after injury and is maintained at 12 weeks. Other recent studies have also demonstrated the beneficial effects of inhibiting β-arrestin signaling using knockout (KO) mouse models. In a model of bleomycin-induced lung fibrosis, loss of β-arrestin1 or 2 resulted in protection from mortality, inhibition of matrix deposition, and improved lung function [21]. Fibrosis was prevented in this model and isolated lung fibroblasts failed to invade the ECM and had altered expression of genes involved in matrix production and degradation. β-arrestin deficiency has also been shown to be beneficial post-MI in global β-arrestin1 KO mice [22]. These mice had increased survival, decreased infarct size, less apoptosis and adverse remodeling compared with post-MI wild type mice.

Although the role of β-arrestins in receptor uncoupling and internalization is well known, more recent work has revealed that β-arrestins are multifunctional scaffolding proteins that are involved in numerous aspects of G protein-coupled receptor signaling. β-arrestins can mediate signaling independent of classical G protein-mediated signaling, and this may be protective in the heart [23]. β-arrestins have been shown to facilitate mitogenic ERK1/2 signaling by scaffolding Raf, MEK1, and c-Src following activation of several GPCRs [24–26]. They have also been shown to activate downstream cell survival pathways including JNK, p38, MAPK, Akt, and PI3K [27–29]. Arrestin-biased signaling refers to their ability to initiate G protein-independent signaling pathways. The β-blockers alprenolol and carvedilol have been shown to stimulate β-arrestin-mediated EGFR transactivation [30] and β-arrestin-mediated β_1_-adrenergic receptor transactivation of the EGFR has been shown to be cardioprotective in mice subjected to chronic sympathetic stimulation [31].

Our data shows that β-arrestin1 and 2 expression in isolated rat CF is upregulated early post-MI and that knockdown of either subtype results in decreased α-SMA and collagen I expression. Baseline and TGF-β-stimulated collagen synthesis were also significantly decreased in these CF after β-arrestin knockdown. These findings suggest that chronically upregulated β-arrestin signaling in fibroblasts is maladaptive and that the beneficial effects seen in other studies may be more specific to cardiac myocytes. Our data provide additional evidence that inhibition of β-arrestins in fibroblasts can reverse the myofibroblast phenotype and inhibit collagen synthesis and fibrosis.

In conclusion, this study provides support for the potential therapeutic role of inhibiting β-arrestin activity following injury in a CF-specific manner to prevent pathological fibrosis and remodeling. The primary mechanisms for inhibition of transformation or activation of quiescent fibroblasts to myofibroblasts and collagen synthesis/fibrosis appear to be restoration of β-AR signaling and intracellular cAMP as well as decreased β-arrestin signaling. Future studies with *in vivo* fibroblast-specific inhibition of β-arrestins will provide further insight into the role of these signaling molecules in ECM remodeling and the progression to HF.
